# AMPK Signaling Axis-Mediated Regulation of Lipid Metabolism: Ameliorative Effects of Sodium Octanoate on Intestinal Dysfunction in Hu Sheep

**DOI:** 10.3390/biom15050707

**Published:** 2025-05-12

**Authors:** Huimin Zhang, Shuo Yan, Zimeng Ma, Ruilin Du, Xihe Li, Siqin Bao, Yongli Song

**Affiliations:** 1 Research Center for Animal Genetic Resources of Mongolia Plateau, College of Life Sciences, Inner Mongolia University, Hohhot 010020, China; zhanghuimin205@gmail.com (H.Z.); yanshuo202504@gmail.com (S.Y.); zimengma68@gmail.com (Z.M.); rallingdu@gmail.com (R.D.); lixh@imu.edu.cn (X.L.); 2 The State Key Laboratory of Reproductive Regulation and Breeding of Grassland Livestock, College of Life Sciences, Inner Mongolia University, Hohhot 010020, China; 3 Inner Mongolia Saikexing Institute of Breeding and Reproductive Biotechnology in Domestic Animal, Hohhot 011517, China

**Keywords:** Hu Sheep, Cadmium, sodium octanoate, Glycerophospholipid metabolism, AMPK signaling pathway

## Abstract

At the present stage, heavy metal pollution, led by environmental exposure to cadmium (Cd), has caused incalculable losses in animal husbandry. The potential value of caprylic acid as a medium- and long-chain fatty acid with a unique role in regulating lipid metabolism has attracted much attention. Our previous study found that octanoic acid levels were significantly reduced under Cd-exposed conditions in Hu Sheep, on the basis of which we investigated the protective effect of sodium octanoate, a derivative of octanoic acid, against Cd exposure in Hu Sheep in the present study. In this study, an animal model of Cd exposure in Hu Sheep was established. Comprehensive assessment of Cd-induced intestinal injury using hematoxylin and eosin (H&E) staining, immunostaining and carried out in-depth analyses combined with lipid metabolomics and transcriptomics. The results showed that Cd exposure triggered intestinal inflammation, barrier function damage and oxidative stress imbalance. Lipid metabolomics analysis showed that Cd exposure severely disrupted lipid metabolic processes, especially the glycerophospholipid metabolic pathway, suggesting that lipid metabolic disorders are closely related to intestinal injury. Notably, sodium octanoate could partially reverse the lipid metabolism abnormality by regulating the Adenosine 5′-monophosphate (AMP)-activated protein kinase (AMPK) signaling pathway, effectively alleviating the Cd toxicity, which provides a brand-new prevention and control strategy for Cd-induced intestinal injury in the livestock industry pollution-mediated disease.

## 1. Introduction

Lipid metabolism, a fundamental biological process involving digestion, absorption, synthesis and catabolism of lipids, plays a pivotal role in maintaining cell homeostasis and systemic physiological functions [1,2,3]. In livestock production, particularly in ruminants such as cattle and sheep, its regulatory mechanisms are critically linked to nutrient efficiency and metabolic health [4,5]. As the core of growth and development, diseases caused by abnormal lipid metabolism are “fatal”, directly threatening the survival rate, growth performance and economic value of livestock [6,7]. Due to the complexity of the physiological and biochemical processes of lipid metabolism and the diversity of influencing factors, the pathogenesis of diseases caused by lipid metabolism abnormalities is not clear, which has led to a great deal of resistance to the prevention and treatment of these diseases [8,9].

As a representative ruminant species, sheep development is critically modulated by a nexus of biotic and abiotic factors, among which anthropogenic environmental pollutants have a direct and significant regulatory role [10,11]. Accompanied by the intensification of industrialization, heavy metal pollution has penetrated into all aspects of the ecological environment, among which Cd environmental exposure is particularly prominent [12,13]. Existing studies on Cd accumulation-induced damage have focused on inflammation-related factors, but the effects on metabolic regulatory networks have not been fully elucidated [14,15,16]. It is worth noting that the homeostatic balance of fatty acids, which are important components of lipid metabolism (including short-chain, medium-chain and long-chain fatty acids), is closely related to the gut microbiota [17,18,19]. Recent studies have shown that environmental factors induced intestinal flora disorders can significantly weaken the resistance and repair ability of sheep to intestinal diseases by decreasing the fatty acid content [20]. In particular, the medium- and long-chain fatty acid sodium octanoate (SO) has emerged as a potential therapeutic agent due to its unique dual role in lipid metabolism regulation and intestinal microbiota modulation [21]. Numerous studies have confirmed that SO has an important role in regulating the body’s metabolic balance, maintaining cellular microenvironmental homeostasis and other biological processes, for example, SO reduces fat deposition, lowers oxidative stress, and maintains intestinal homeostasis [22,23,24].

In a previous study, Cd exposure was demonstrated to significantly impair Hu Sheep growth and development, characterized by severe weight loss and persistent digestive and absorption dysfunction [20]. This observation prompts us to hypothesize that Cd exposure may contribute to intestinal pathogenesis through interference with lipid metabolic pathways. It is of interest to note that, unlike other etiologic mechanisms, abnormal lipid metabolism in sheep is directly related to their growth rate, disease resistance, and even breed selection [25,26]. Different breeds of sheep have significant differences in disease resistance and disease recovery [27,28]. We chose Hu Sheep with strong disease resistance characteristics as the target of our study, aiming to analyze the intrinsic correlation between the occurrence of intestinal diseases and lipid metabolism abnormality in Cd-exposed to Hu Sheep, reveal its molecular mechanism, and explore the potential therapeutic drugs.

## 2. Materials and Methods

### 2.1. Materials and Reagents

Cadmium chloride (CdCl_2_) and sodium octanoate were procured from Shanghai Macklin Biochemical Technology Co., Ltd., Shanghai, China. Commercial ELISA kits for cytokine detection (including IL-6, IL-1β, TNF-α) were obtained from Boyan Biotechnology Co., Ltd., Nanjing, China. Additional biochemical assay kits measuring total superoxide dismutase (SOD) activity, catalase (CAT) levels, glutathione (GSH) content, and lipid peroxidation markers (malondialdehyde, MDA) were supplied by Beyotime Biotechnology, Shanghai, China.

### 2.2. Animals and Experimental Design

Eighteen (Two months old, 20–22 kg) healthy male Hu Sheep were provided by Shengle Technology Co., Ltd., Hohhot, China. After 7 days of acclimatization, the sheep were randomly assigned to three experimental groups: (1) The control (CON) group received sterile saline; (2) Cd group received 20 mg/kg CdCl_2_ per day by oral gavage (protocol adapted from Li et al. [29]); (3) SO group received a combined intervention of 20 mg/kg CdCl_2_ + 5 mg/kg sodium octanoate. All groups have been submitted to oral gavage. All sheep were slaughtered on day 22 after modeling. Samples were collected from each sheep immediately after slaughter. Jejunal tissue was rinsed and immediately stored at −80 °C until further analysis.

### 2.3. H&E Staining

The severity of jejunal lesions was scored macroscopically and histologically. Jejunal samples were sequentially fixed (4% paraformaldehyde), paraffin-embedded and sectioned for preparation. Sections were deparaffinized twice (10 min each) by xylene, followed by sequential rehydration in a gradient of 100%, 95%, 85%, and 75% ethanol for 3 min each, and immersion in distilled water for 2 min. Hematoxylin staining was performed for 5 min followed by water washing to remove floating color, differentiation in differentiation solution for 30 s, rinsing under running water twice (3 min each), then staining with eosin for 1 min, rapid rehydration through a gradient of 75%, 85%, 95%, and 100% ethanol, dimethylbenzene clearing twice (1 min each), and sealing of the film by neutral resins. Histologic analysis was performed by capturing images with a 10× magnification imaging system to assess the extent of inflammation and mucosal/cryptal damage.

### 2.4. Enzyme-Linked Immunosorbent Assay

The jejunal levels of IL-6, IL-1β and TNF-α were quantified using ELISA kits using at least three biological replicates. 0.1 g of jejunal tissue was cut and added to 0.9 mL of phosphate-buffered saline (PBS) solution and homogenized in an ice bath, and the supernatant was centrifuged at 5000× *g* for 10 min. After the kit components were returned to room temperature, the working solution was prepared. Standard wells (50 µL of 160, 80, 40, 20, 10, 5 pg/mL gradient standards were added), 0-value wells (50 µL of sample diluent), sample wells (50 µL of the sample to be tested), and blank wells (no sample was added) were set up in the enzyme plate. Except for the blank wells, 100 µL of horseradish peroxidase (HRP)-labeled antibody was added to each well, and incubated for 60 min at 37 °C, protected from light. After incubation, the solution was discarded and patted dry, and each well was filled with washing solution and left to stand for 20 s, then shaken off, and the washing was repeated five times. Add an equal volume of mixed substrate A/B solution, incubate at 37 °C for 15 min, avoiding light., and terminate the reaction by adding 50 µL of termination solution to each well, and read the absorbance value at 450 nm.

Total Superoxide Dismutase Assay: Weigh 0.1 g of jejunal tissue and add 1 mL of SOD sample preparation solution, the mixture is homogenized on ice and then centrifuged at 12,000× *g* for 5 min (4 °C) before collecting the supernatant. The WST-8/enzyme working solution was prepared according to the ratio of 151 µL of assay buffer, 8 µL of WST-8, and 1 µL of enzyme solution per well. 40× reaction initiation solution was diluted 40-fold with assay buffer for spare parts, and the standards were diluted with the SOD sample preparation solution to a gradient of 100, 50, 20, 10, 5, 2, and 1 U/mL, and all the reagents were stored in an ice bath. In a 96-well plate, set up standard wells (20 µL of different concentrations of standards), blank wells, and sample wells (20 µL of samples to be tested), add 160 µL of WST-8/enzyme working solution and 20 µL of diluted reaction initiation solution to each well, and then measure the absorbance at 450 nm after incubation at 37 °C away from light for 30 min.

Catalase assay: 0.1 g of jejunal tissue was homogenized in an ice bath with 1 mL of lysate, and the supernatant was collected after centrifugation at 12,000× *g* for 10 min (4 °C). The stock solution of Hydrogen peroxide (H_2_O_2_) (≈1 M) was diluted to 10 mM, and 250 mM and 5 mM H_2_O_2_ working solutions were prepared by calibrating with UV spectrophotometry (240 nm), and the working solution for color development was prepared by mixing catalase and color-developing substrate in the ratio of 1:1000. When the standard curve was established, 0, 12.5, 25, 50, 75 µL of 5 mM H_2_O_2_ solution was taken, and the assay buffer was added to a final volume of 100 µL (corresponding to the final concentration of 0, 0.625, 1.25, 2.5, 3.75 mM), 4 µL of each different concentrations of the prepared standard solutions were transferred to a 96-well plate, and 200 µL of the color working solution was added, and then the plate was incubated at 25 °C and protected from light for 15 min, and the absorbance was measured at 520 nm. For sample detection, 7.5 µL of sample was mixed with 32.5 µL of assay buffer, and 10 µL of 250 mM H_2_O_2_ solution was added to start the reaction (25 °C, 3 min), and then the reaction was terminated by the addition of 450 µL of termination solution. After 10 µL of the termination solution was mixed with 40 µL of assay buffer, 10 µL of the 50 µL system from the previous step was added to one well of a 96-well plate, followed by 200 µL of color development working solution was added, and the reaction was incubated for 15 min at 25 °C, protected from light, and the absorbance was finally measured at 520 nm.

Lipid Peroxidation MDA Assay: Take 0.1 g of jejunal tissue and add 1 mL of lysis solution to ice bath homogenization, centrifuge at 12,000× *g* for 10 min (4 °C) and take the supernatant. TBA powder was dissolved in a special diluent to make a 0.37% solution, heated at 70 °C and vortexed until completely dissolved. The standards were diluted with distilled water to a series of concentrations of 1, 2, 5, 10, 20 and 50 µM. A blank control (0.1 mL PBS), a standard curve group (0.1 mL of different concentrations of standards), and a sample group (0.1 mL of samples to be tested) were set up for the assay. 0.2 mL of MDA assay working solution was added to each group, mixed well, and then heated in a boiling water bath at 100 °C for 15 min. After cooling to room temperature, the sample was centrifuged at 1000× *g* for 10 min and 200 µL of supernatant was transferred to a 96-well plate, and the absorbance was measured at 532 nm.

Reduced Glutathione Assay: Use 10 mM GSSG stock solution (5 mg GSSG + 816 µL Milli-Q water) and dilute with Protein Removal Reagent M (0.2 g Protein Removal Reagent M + 4 mL buffer) in 15, 10, 5, 2, 1 and 0.5 µM gradients. Add 0.1 g of jejunal tissue to 300 µL of Reagent M, grind thoroughly, allow to stand at 4 °C for 10 min, centrifuge at 10,000× *g* for 10 min, and extract the supernatant. Take 100 µL of sample supernatant, add 20 µL of diluted GSH Scavenging Auxiliary Solution (47 µL water + 53 µL Auxiliary Solution), vortex to mix, then add 4 µL of GSH Scavenging Reagent Workup (10.8 µL reagent + 89.2 µL ethanol) and react for 60 min at 25 °C to completely remove free GSH. In a 96-well plate, add a blank (10 µL of Reagent M), a standard (10 µL of gradient solution), and a sample (5 µL of supernatant + 5 µL of Reagent M), each mixed with 150 µL of Total Glutathione Assay Work-up (6.6 µL of diluent enzyme + 6.6 µL of 5,5′-Dithiobis-(2-nitrobenzoic acid) (DTNB) + 150 µL of buffer), and incubate for 5 min at 25 °C. Then, 50 µL of 0.5 mg/mL Nicotinamide Adenine Dinucleotide Phosphate Hydrogen (NADPH) working solution (10 µL stock solution + 790 µL buffer) was added and absorbance was read immediately at 412 nm. Absorbance was measured every 5 min until 25 min.

### 2.5. Immunofluorescence Staining

Jejunal tissue sections were dewaxed by xylene 1 and xylene 2 for 15 min each, dehydrated by graded ethanol (anhydrous ethanol 1, anhydrous ethanol 2, 95% ethanol, 85% ethanol, 75% ethanol for 5 min each) and washed in water; antigenic repair was then performed, and routine tissues were repaired by autoclaving (1× citrate buffer, pH 6.0, high pressure bubbling and repaired for 2 min); repaired sections were blocked for 20 min by 3% H_2_O_2_, endogenous enzymes were blocked for 20 min at room temperature, and 10% homologous serum (e.g., goat serum) was blocked for 30 min at 37 °C; the primary antibody was diluted 1:200 and incubated overnight at 4 °C or for 2 h at 37 °C, and the HRP-labeled secondary antibody was incubated for 1 h at 37 °C, followed by a dropwise addition of the TYR-488 reagent for a 30-min reaction at 37 °C, and washed 3 times with PBS; the sections were again subjected to microwave antigen repair (1× citrate buffer, high fire for 5 min), optional secondary serum closure, repeat the steps of primary antibody and secondary antibody incubation, and replace the TYR-555 TSA reagent 37 °C incubation for 30 min; 4′,6-Diamidino-2-phenylindole dilactate (DAPI) staining of the nucleus for 5 min, PBS washed. Finally, the mounting of slides was carried out by means of an Antifade Mounting Medium with DAPI (H-1200, VECTASHIELD, Burlingame, CA, USA) and imaged by confocal microscopy using a Nikon Ax upright laser confocal microscope (SMZ7457, Nikon, Tokyo, Japan) and the Nikon operating software (NIS Elements Viewer, Version 5.21.00).

### 2.6. Immunohistochemical Staining

Thermal antigen recovery was performed during immunohistochemical processing by microwave heating for 20 min using 0.01 M citrate buffer pH 6.0/1 mM EDTA pH 8.0. After cooling to room temperature, tissue sections were pretreated sequentially: endogenous peroxidase was quenched with 3% H_2_O_2_ (10 min), serum was blocked at ambient temperature (60 min), and the primary antibody was then incubated at 4 °C overnight. Colors were developed using the ABC Peroxidase Detection System (Vector Labs) in combination with Diaminobenzidine (DAB), and the counterstain was hematoxylin. Images were taken using a Nikon inverted microscope stage (model SMZ7457). Antibody dilution concentrations are shown in Table S1 of the Supplementary Material.

### 2.7. Quantitative RT-PCR Analysis

Total RNA extraction was performed using the RNeasy Mini Kit (Qiagen, 74104, Hilden, Germany) following a standardized protocol. The GoScript™ Reverse Transcription System (Promega, A5001, Singapore) was used to generate cDNA. real-time quantitative PCR analysis was performed on a LightCycler 96 platform (Roche, Basel, Switzerland) using KAPA SYBR FAST chemistry (KR0389) with at least three biological replicates, and all results were similar. Relative transcript levels were assessed using the 2^−ΔΔCt^ method with GAPDH as the normalization reference. Primer sequence specifications are shown in Supplemental Table S2.

### 2.8. RNA-Seq Analysis

Transcriptome analysis of jejunal tissue specimens from each group was performed using RNA sequencing. Total RNA was extracted using TRIzol reagent following the steps provided by the manufacturer. cDNA libraries were constructed using cDNA library construction and bioinformatics analysis, and high-throughput sequencing was performed on the Illumina NovaSeq6000 platform. Gene expression levels were estimated by calculating fragment values. Exons per one thousand nucleotides were mapped to one million reads (FPKM). We selected genes with a fold change greater than 2 and a *p*-value less than 0.05. Differentially expressed genes (DEGs) were functionally annotated by Kyoto Encyclopedia of the Genes and Genome (KEGG) pathway enrichment analysis, and quantitative metrics were expressed in units of reads per kilobase per million.

### 2.9. Broadly Targeted Lipidomics

This study employed liquid chromatography-electrospray ionization tandem mass spectrometry (LC-ESI-MS/MS) for broadly targeted lipidomics analysis. Tissue samples were homogenized in liquid nitrogen and extracted using methyl tert-butyl ether/methanol (3:1, *v*/*v*) containing internal standards, followed by phase separation with water and centrifugation. The organic layer was concentrated and reconstituted in acetonitrile/isopropanol (1:1, *v*/*v*) for analysis. Chromatographic separation was performed on a Thermo Accucore™ C30 column (2.1 × 100 mm, 2.6 µm) with an acetonitrile-isopropanol/water gradient containing 0.1% formic acid and 10 mM ammonium formate. Mass spectrometric detection was conducted using a QTRAP^®^ 6500+ system (Sciex, Framingham, MA, USA) in both positive and negative ionization modes, with ion spray voltages of ±5500/4500 V, source temperature at 500 °C, and gas parameters optimized for sensitivity. Dynamic multiple reaction monitoring (MRM) methods were implemented for lipid quantification, supported by commercial lipid standards for system calibration and quality control.

### 2.10. Bioinformatics Analysis

Multivariate analysis was performed by principal component analysis (PCA) using the prcomp algorithm in the R Statistical Suite. The data were subjected to unit variance scaling before unsupervised PCA.

Hierarchical cluster analysis and Pearson correlation coefficients: ① Hierarchical clustering of metabolite profiles was performed by heatmaps of dendrograms; ② Inter-sample correlation matrices were generated by Pearson coefficient calculations (cor function). Both HCA and PCC were implemented with the pheatmap package, and the metabolite intensities were standardized by ANOVA normalization to visualize the comparisons.

### 2.11. KEGG Annotation and Enrichment Analysis

The identified metabolites were annotated using the KEGG compound database (http://www.kegg.jp/kegg/compound/, accessed on 2 March 2025), and the annotated metabolites were subsequently mapped to the KEGG pathway database (http://www.kegg.jp/kegg/pathway.html, accessed on 2 March 2025). Pathways with significantly differentially expressed metabolites were then subjected to metabolite set enrichment analysis (MSEA), and their significance was determined by hypergeometric test *p* values.

### 2.12. Statistical Analysis

In this study, GraphPad Prism 7.0 (GraphPad, La Jolla, CA, USA) or the R software package (4.5.0) was used for data analysis and processing. All data were presented as the mean ± standard deviation (SD). For statistical analysis, the t-test was employed for comparisons between two groups, while one-way analysis of variance (ANOVA) was used for comparisons among multiple groups. The criterion for determining whether the difference was statistically significant was *p* < 0.05. Different levels of significance were marked as follows: (* *p* < 0.05; ** *p* < 0.01; *** *p* < 0.001).

## 3. Results

### 3.1. Sodium Octanoate Ameliorates Cadmium-Induced Intestinal Inflammation in Hu Sheep

**Figure 1:**
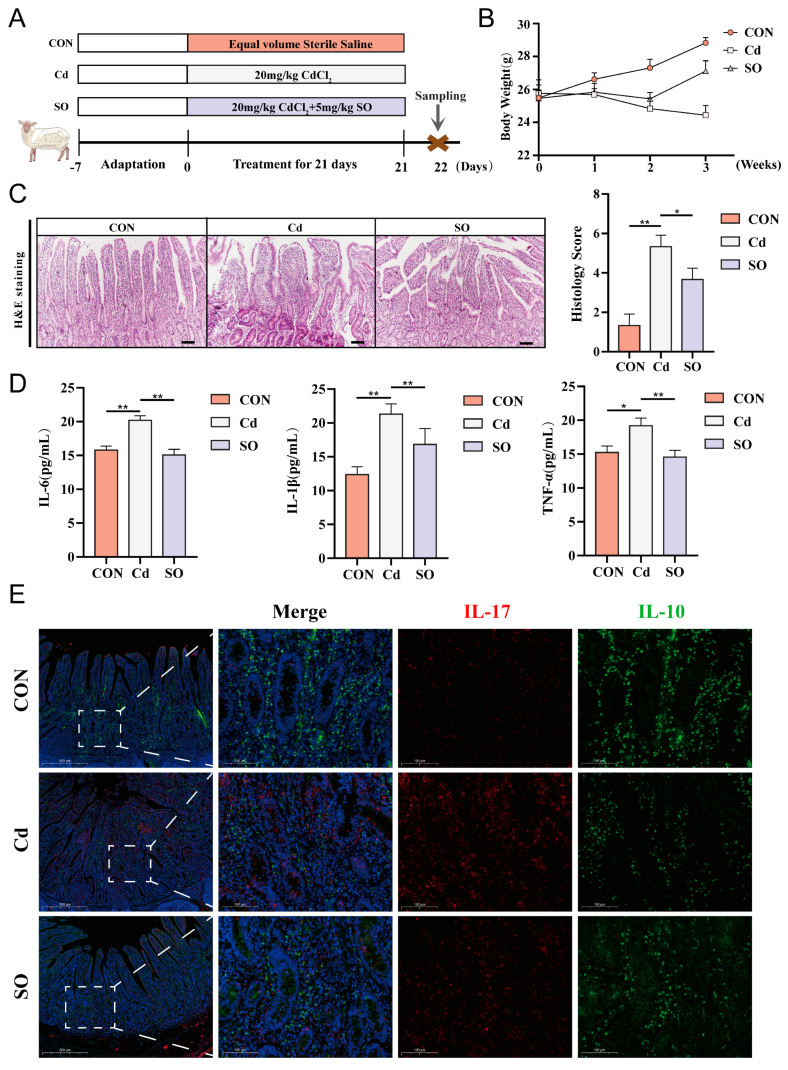
Sodium octanoate ameliorates Cadmium-induced intestinal inflammation in Hu Sheep. (**A**) Experimental design for this study (n = 6). (**B**) Changes in body weight during experiment periods. (**C**) H&E staining results and histologic scoring of jejunal tissue (10×). (**D**) Concentrations of IL-6, IL-1β, and TNF-α in the jejunum. (**E**) Immunofluorescence images of three groups of jejunum stained for IL-17 and IL-10 (10×, 40×). All the above experiments were repeated three times independently and the data were expressed as “Mean ± standard deviation (SD)” (* *p* < 0.05, ** *p* < 0.01).

Eighteen two-month-old Hu Sheep (20–22 kg) were randomly divided into three groups. These included CON group, Cd group, and SO group. Modeling was started after one week of acclimatization. During the modeling period, the CON group was given saline solution by gavage, the Cd group was given 20 mg/kg CdCl_2_ every morning, and the SO group was supplemented with 5 mg/kg sodium octanoate daily in the afternoon on the basis of the same Cd exposure. The modeling period was 21 days and samples were collected after euthanasia on day 22 (Figure 1A). During the modeling period, we monitored and recorded the body weights of the three groups daily, and we found that the body weights of the CON group showed a steady increase. On the contrary, the body weights of the Hu Sheep in the Cd group showed a decreasing trend throughout the modeling process. The body weights of the SO group were the same as those of the Cd group at the beginning of the modeling period, but after one week, the body weights of the SO group were significantly higher than those of the Cd group, and from the third week onward, the body weights of the SO group increased significantly, and the treatment effect was very obvious, even though it did not return to the basal body weights of the CON group (Figure 1B). Subsequently, the jejunal tissues of the three groups were fixed, embedded, sectioned. Hematoxylin and eosin staining showed that the degree of jejunal damage and histopathological scores were higher in the Cd group than in the CON group, whereas supplementation with SO restored the crypts’ depth, alleviated inflammatory cell infiltration to a certain extent, and the histopathological scores were also restored (Figure 1C). Next, we assessed the expression levels of inflammatory cytokines in jejunal tissues by ELISA assay. It was found that the expression levels of IL-6, IL-1β and TNF-α were significantly elevated in the Cd group compared to the CON group. Sodium octanoate treatment alleviated this elevation to varying degrees (Figure 1D). Subsequent immunofluorescence double-staining analyses of IL-17 and IL-10 showed that Cd exposure resulted in a significantly increased in the number of positive cells for the pro-inflammatory cytokine IL-17 and a significantly decreased in the number of positive cells for the anti-inflammatory cytokine IL-10 (Figure 1E). Notably, these pathologic changes were effectively ameliorated by SO treatment, suggesting the protective potential of SO against Cd-induced inflammatory. In conclusion, these findings suggest that SO may attenuate Cd-induced intestinal injury by protecting epithelial integrity and restoring inflammatory balance.

### 3.2. Cadmium-Induced the Oxidative Stress Disrupts Intestinal Barrier Integrity and the Reparative Effect of Sodium Octanoate

**Figure 2:**
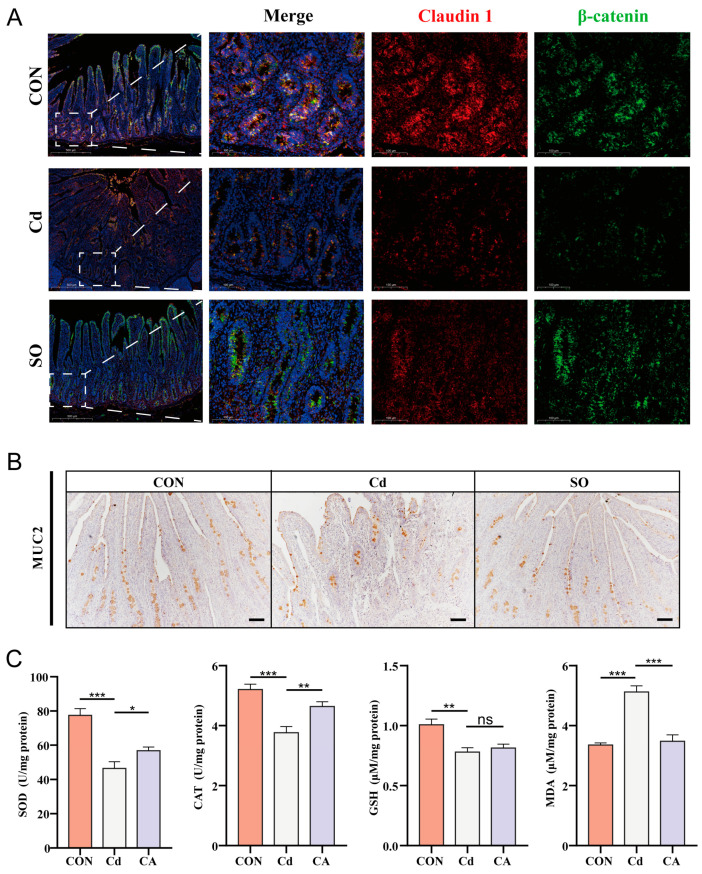
Cadmium-induced the oxidative stress disrupts intestinal barrier integrity and the reparative effect of sodium octanoate. (**A**) Immunofluorescence images of three groups of jejunum stained with claudin1 and β-catenin. (**B**) Immunohistochemical analysis of MUC2 in the jejunum of three groups (20×). (**C**) Changes in indicators of oxidative stress in three groups. All the above experiments were repeated three times independently and the data were expressed as “Mean ± standard deviation (SD)” (* *p* < 0.05, ** *p* < 0.01, *** *p* < 0.001).

In order to investigate the damage mechanism of Cd exposure on intestinal barrier integrity and the therapeutic effect of SO, we firstly analyzed the expression of tight junction proteins, claudin1, and β-catenin by immunofluorescence double-staining. The results showed that Cd exposure significantly disrupted the structural integrity of the intestinal barrier, as evidenced by diminished claudin1/β-catenin co-localization; whereas SO treatment restored this trend, suggesting its protective effect on the tight junction structure (Figure 2A). Give that the mucus barrier is the first line of defense against harmful substances in the intestinal lumen, and the secretion status of its core component, MUC2, directly affects intestinal permeability [30]. We further assessed the MUC2 expression by immunohistochemistry. The results showed that the number of MUC2-positive cells was significantly reduced in the Cd group compared with the CON group, and SO treatment significantly increased the number of MUC2-positive cells (Figure 2B). Oxidative stress is an important mechanism of heavy metal toxicity [31], so, we further understood oxidative stress status. The results showed that Cd exposure significantly decreased SOD, CAT, GSH levels, while elevating MDA levels. Notably, SO treatment demonstrated potent restorative efficacy in reversing these abnormal changes (Figure 2C). These data suggest that Cd exposure may lead to disruption of tight junctions by disrupting redox homeostasis, and that the gut-protective effects of SO may be closely related to its antioxidant properties [21].

### 3.3. Sodium Octanoate Attenuates Cadmium-Induced Disruption of Immune Homeostasis

**Figure 3:**
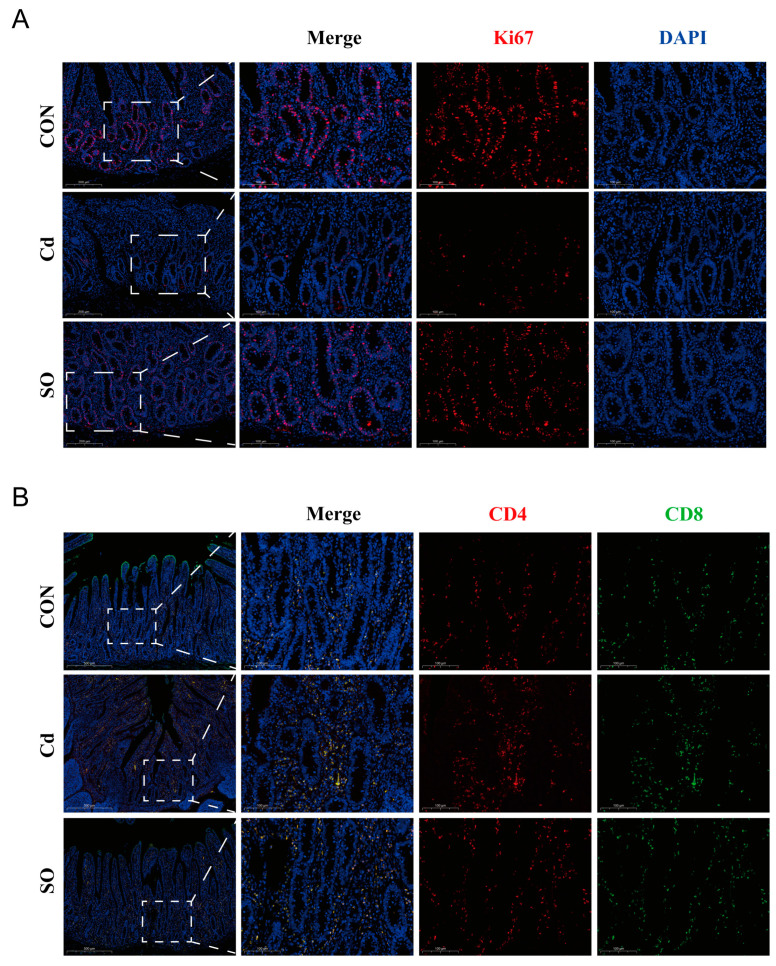
Sodium octanoate attenuates cadmium-induced disruption of immune homeostasis. (**A**) Immunofluorescence images of Ki67 staining of three groups of jejunum (20×, 40×). (**B**) Immunofluorescence images of three groups of jejunum stained for CD4 and CD8 (10×, 40×).

To systematically assess the multifaceted effects of Cd exposure on intestinal homeostasis, we further explored its regulatory effects on epithelial regenerative capacity and immune microenvironment. Continuous regeneration of intestinal epithelial cells is a central mechanism of barrier repair [32], we examined the proliferative activity of epithelial cells by Ki67 immunofluorescence staining. The results showed that the number of Ki67-positive cells was significantly reduced in the Cd group compared with the CON group, suggesting that Cd significantly inhibited the proliferation of epithelial cells; whereas SO treatment partially restored the number of Ki67-positive cells (Figure 3A). Notably, disruption of the intestinal barrier is often accompanied by hyperactivation of the immune system [33]; therefore, we analyzed lymphocyte infiltration in the lamina propria by CD4/CD8 immunofluorescence staining, the result demonstrated that CD4^+^ and CD8^+^ T-cell populations were significantly increased in the Cd group compared with the CON group, suggesting that Cd exposure may disrupt immune function and promote inflammation occurrence. Strikingly, SO treatment reversed this trend, significantly reducing CD4^+^ and CD8^+^ T-cell infiltration, which underscores the potential function of SO to restore immune homeostasis (Figure 3B).

### 3.4. Sodium Octanoate Alleviates Lipid Metabolism Disorders Induced by Cadmium Exposure

**Figure 4:**
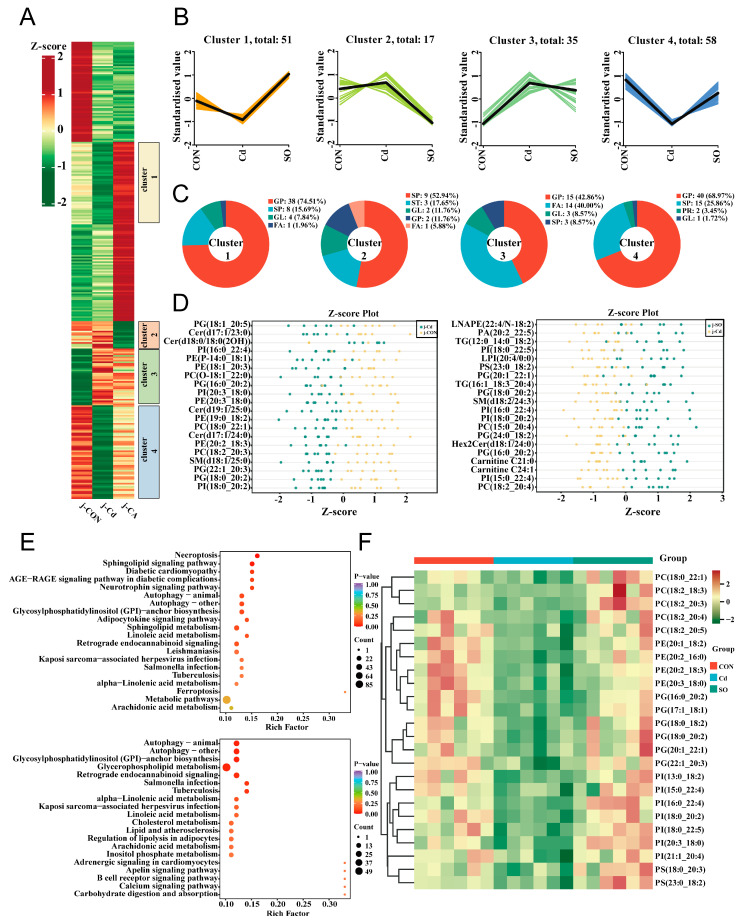
Sodium octanoate broadly regulates jejunal lipid metabolism. (**A**) DALs were clustered and plotted as a heatmap. (**B**) Four clusters (clusters 1–4) were highlighted, and the trend lines indicating the change in levels in the jejunum of these DALs were shown. (**C**) DALs in each cluster were subcategorized based on their lipid classes. (**D**) Distribution of the top 20 DALs in the CON group compared to the Cd group and the Cd group compared to the SO group. (**E**) KEGG enrichment analysis of DALs in CON vs. Cd group and Cd vs. SO group. (**F**) Heat map of DALs associated with Glycerophospholipid metabolism.

Disturbed lipid metabolism is a critical pathological feature of Cd-induced toxicity, to elucidate this mechanism, we performed untargeted lipidomic sequencing and hierarchical cluster analysis, which subdivided all differentially altered lipid metabolites (DALs) into 6 clusters based on alteration patterns, and 4 clusters of interest were labeled in (Figure 4A). As shown in (Figure 4B), the relative contents of DALs were significantly up-regulated in clusters 1 and 4 of the SO group compared to the Cd group. Notably, the contents of DALs in both cluster 2 and cluster 3 were significantly up-regulated after Cd exposure and reversed after SO supplementation. We then found that glycerophospholipid (GP) had the highest percentage in clusters 1, 3, and 4, while sphingomyelin (SP) had the highest abundance in cluster 2 (Figure 4C). Next, we screened the top 20 enriched DALs in the CON versus Cd and Cd versus SO groups and found that Cd exposure resulted in abnormally low levels of the differential lipid metabolites PI (16:0_22:4), PG (16:0_20:2), PG (18:0_20:2), and PI (18:0_20:2) as compared with those in the CON group, but supplementation with SO were significantly restored the levels of these metabolites (Figure 4D). Next, we analyzed KEGG enrichment of DALs in three groups of Hu Sheep. The results showed that the DALs in the CON and Cd groups were significantly enriched in the Sphingolipid signaling pathway, whereas the DALs in the Cd and SO groups were significantly enriched in the Glycerophospholipid metabolic pathway (Figure 4E). We further constructed a heat map of DALs enrichment of glycerophospholipid metabolic pathway and found that these DALs were significantly down-regulated in the Cd group, while the DALs expression levels were restored after SO supplementation (Figure 4F). These findings suggest that SO ameliorates Cd-induced lipid metabolism disorders through regulating the homeostasis of glycerophospholipid metabolic pathway.

### 3.5. Sodium Octanoate Broadly Modulates the Jejunal Transcriptional Profile

**Figure 5:**
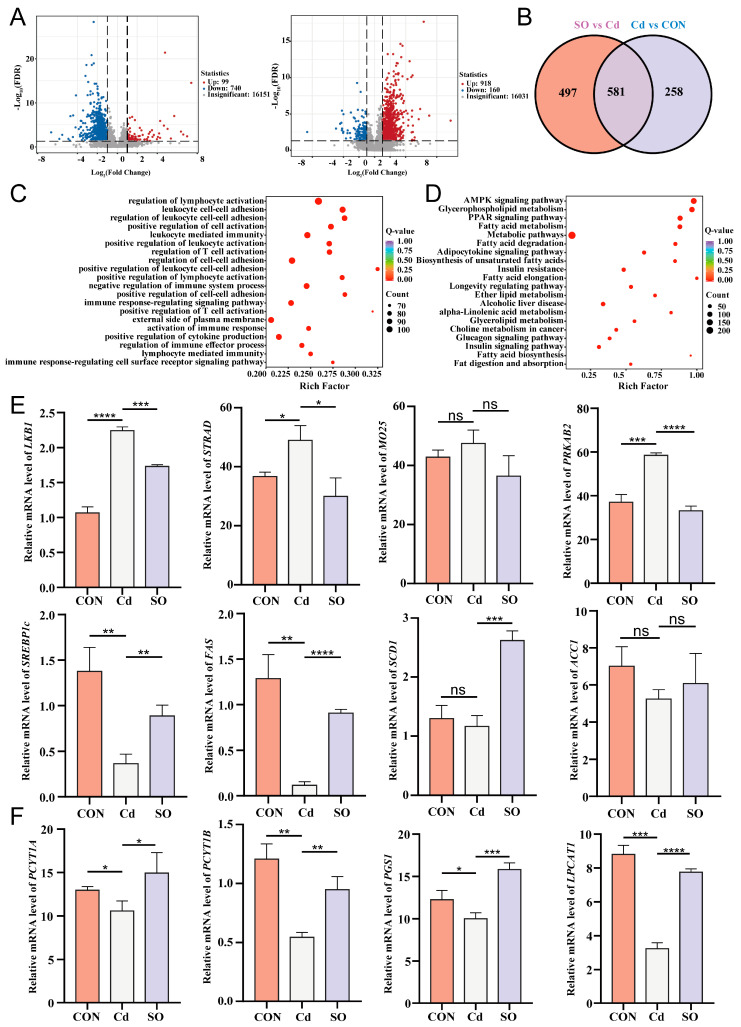
Sodium octanoate broadly modulates the jejunal transcriptional profile. (**A**) Volcano plots of DEGs (CON vs. Cd, Cd vs. SO). (**B**) Venn diagram of DEGs in the CON group vs. Cd group and Cd group vs. SO group. (**C**) GO enrichment analysis of DEGs in the Cd and SO groups. (**D**) KEGG enrichment analysis of DEGs in the Cd and SO groups. (**E**) *LKB1*, *STRAD*, *MO25*, *PRKAB2*, *SREBP1c*, *FAS*, *SCD1* and *ACC1* gene expression levels in the three groups. (**F**) *PCYT1A*, *PCYT1B*, *PGS1* and *LPCAT1* gene expression levels in the three groups. All the above experiments were repeated three times independently and the data were expressed as “Mean ± standard deviation (SD)” (* *p* < 0.05, ** *p* < 0.01, *** *p* < 0.001, **** *p* < 0.0001).

To further explore the therapeutic mechanism of SO, we performed transcriptome sequencing of jejunal tissues from Hu Sheep among three groups. The volcano map showed that 99 DEGs were up-regulated and 740 DEGs were down-regulated in the Cd group compared with the CON group. There were 918 DEGs up-regulated and 160 DEGs down-regulated in the SO group compared with the Cd group (Figure 5A). The Venn diagram shows 258 unique DEGs in the Cd vs. CON comparison and 497 in the SO vs. Cd comparison. In addition, 581 DEGs are common to both comparisons (Figure 5B). To further investigate the reason for this phenomenon, we performed gene ontology (GO) enrichment analysis of DEGs in the Cd and SO groups of Hu Sheep. The results showed that the DEGs were mainly enriched for the terms of regulation of lymphocyte activation, leukocyte-mediated immunity, regulation of cell-cell adhesion, leukocyte cell-cell adhesion and other processes (Figure 5C). We next performed KEGG enrichment analysis of DEGs in the Cd and SO groups of Hu Sheep. The results showed that the AMPK signaling pathway was the most significantly enriched pathway among the DEGs (Figure 5D). Adenosine 5′-monophosphate (AMP)-activated protein kinase is a core regulator of cellular energy metabolism, which mainly responds to changes in energy status (e.g., decrease in ATP/AMP ratio) and maintains energy homeostasis by regulating various metabolic pathways. In lipid metabolism, AMPK plays a key regulatory role by inhibiting lipid synthesis and promoting lipolysis and oxidation. We detected gene expression changes at key nodes of the pathway by qPCR. The results showed that Cd exposure up-regulated the expression of AMPK activation complex-related genes *LKB1*, *STRAD*, and *MO25*, suggesting that Cd may activate AMPK signaling through the *LKB1-STRAD-MO25* complex. Notably, the AMPK regulatory subunit gene *PRKAB2* and the catalytic subunit genes *PRKAG1*, *PRKAG2*, *PRKAG3* were both significantly up-regulated in the Cd group, and this multisubunit synergistic expression phenomenon may enhance the stability of the AMPK complex. Meanwhile, the key genes for fatty acid oxidation, *CPT1* (carnitine palmitoyltransferase1) and *MCD* (malonyl coenzyme A decarboxylase), also showed up-regulation, suggesting that AMPK promotes mitochondrial fatty acid β-oxidation by deregulating malonyl coenzyme A inhibition of CPT1. In terms of lipid synthesis regulation, we observed reduced expression of key lipogenic genes *SREBP1c* (sterol regulatory element-binding protein 1c), *FAS* (fatty acid synthase), *SCD1* (stearoyl coenzyme A desaturase 1), and *ACC1* (acetyl coenzyme A carboxylase 1) in the Cd group, suggesting that AMPK inhibits *ACC1* activity through phosphorylation and thus down-regulates the cascade of *SREBP1c*, *SCD1* and *FAS*. This bidirectional regulation of fat synthesis inhibition and catabolism enhancement reveals the central role of the AMPK pathway in Cd-induced energy metabolic remodeling. Interestingly, after SO treatment, all of the above gene expressions showed reversal, suggesting that SO may restore the dynamic balance of lipid metabolism by regulating AMPK activity (Figure 5E, Figure S1A). Further analysis showed that Cd exposure also resulted in decreased expression of *PCYT1A*, *PCYT1B*, *PGS1*, *LPCAT1*, *CDS2*, *CEPT1*, *LPIN1*, *LPIN2* and *LPCAT2* genes (Figure 5F, Figure S1B). These genes encode glycerophospholipid, whose down-regulation disrupts membrane lipid raft integrity and fluidity. Whereas SO treatment significantly restored the expression of these genes, these results suggest that the AMPK pathway may be involved in the Cd toxicity repair process by regulating membrane lipid synthesis.

### 3.6. A Potential Link Between Lipid Metabolism and Transcriptional Regulation

**Figure 6:**
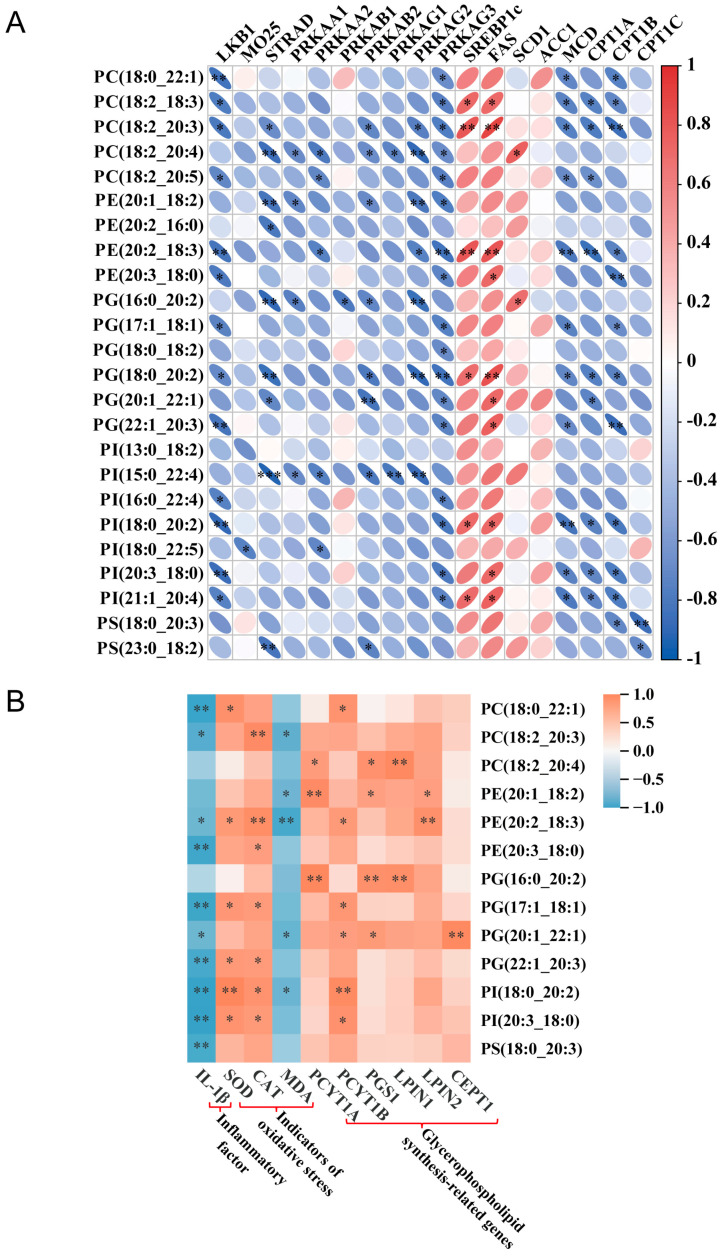
Correlation analysis. (**A**) Correlation analysis of DALs and DEGs. (**B**) Correlation analysis of inflammatory factors, oxidative stress indicators, lipid synthases, and DALs (* *p* < 0.05, ** *p* < 0.01, *** *p* < 0.001).

In order to further analyze the role of lipid metabolism in Cd-induced intestinal injury and the therapeutic effect of SO in Hu Sheep, we conducted a comprehensive analysis combining lipidomics and transcriptomics. The results showed that there were significant correlations between key regulatory genes of the AMPK signaling pathway and specific phospholipid species: *LKB1* was significantly negatively correlated with PC (18:0_22:1), PE (20:2_18:3), PG (22:1_20:3), PI (18:0_20:2), PI (20:3_18:0), and *MO25* was significantly negatively correlated with PI (18:0_22:5). *STRAD* was significantly negatively correlated with PC (18:2_20:4), PE (20:1_18:2), PG (16:0_20:0), PG (18:0_20:2), PI (15:0_22:4), and PS (23:0_18:2), with the highest negative correlation with PI (15:0_22:4). *PRKAB2* was significantly negatively correlated with PG (20:1_22:1); *PRKAG3* was significantly negatively correlated with PE (20:2_18:3) and PG (18:0_20:2); *SREBP1c* and *FAS* were significantly positively correlated with PC (18:2_20:3) and PE (20:2_18:3); and *MCD* and *CPT1A* were significantly negatively correlated with PE (20:2_18:3) were significantly negatively correlated (Figure 6A). These results suggest that they are involved in the lipid biosynthesis pathway. Functional correlation analysis further revealed that several phospholipids such as PC (18:0_22:1), PE (20:3_18:0), PG (17:1_18:1), PG (22:1_20:3), PI (18:0_20:2), PI (20:3_18:0), and PS (18:0_20:3) were significantly negatively correlated with the pro-inflammatory cytokine IL-1β. Among the genes related to oxidative stress, PI (18:0_20:2) showed significant positive correlation with SOD, PC (18:2_20:3) and PE (20:2_18:3) showed significant positive correlation with CAT, PE (20:2_18:3) showed significant negative correlation with MDA. Analysis of glycerophospholipid metabolism showed that PG (16:0_20:2) was significantly and positively correlated with glycerophospholipid synthesis-related genes PCYT1A, PGS1 and LPIN1, suggesting that this metabolite plays an important role in regulating lipid metabolism disorders induced by Cd exposure (Figure 6B).

## 4. Discussion

Cadmium, a globally prevalent heavy metal pollutant, induces systemic toxicity through disruption of gut microbiome [16,34,35]. In our Hu Sheep model, Cd exposure caused weight loss, correlating with microbiota dysbiosis, which is consistent with previous reports on the inhibitory effects of Cd on growth and development in livestock [20]. Histopathological analysis of the jejunum showed increased jejunal damage and elevated histopathological scores in the Cd group compared to the CON group. Meanwhile, the levels of inflammatory cytokines (e.g., IL-6, IL-1β and TNF-α) were elevated, indicating the presence of intestinal inflammation. Also, the integrity of the intestinal barrier of Hu Sheep was severely damaged after Cd exposure. Immunofluorescence analysis showed diminished colocalization signals of the tight junction proteins claudin1 and β-catenin, and immunohistochemistry showed a significant reduction in the number of MUC2-positive cells, which are critical for maintaining the mucus barrier [36]. In addition, Cd exposure resulted in abnormal oxidative stress-related indices, with decreased levels of SOD, CAT and GSH and increased levels of MDA, highlighting the role of oxidative stress in Cd-induced toxicity [37].

Notably, the gut microbiota plays a crucial role in maintaining gut health. Cadmium exposure has been shown to alter the composition and function of the gut microbiota [38]. In the present study, Cd exposure may indirectly affect intestinal physiological functions by disrupting the gut microbiota. The intestinal microbiota is closely linked to the lipid metabolism of the host, and they are involved in the metabolism and synthesis of fatty acids, which affects the absorption and utilization of lipids in the host [39]. For example, some beneficial bacteria are able to produce short-chain fatty acids, which not only provide energy to intestinal epithelial cells, but also regulate the expression of genes related to lipid metabolism [40]. When Cd exposure disrupts the balance of the intestinal microbiota, it may lead to a decrease in the number of beneficial bacteria and an increase in the number of harmful bacteria, which in turn interferes with lipid metabolism processes [20]. This may be a potential mechanism by which Cd exposure triggers lipid metabolism disorders.

Importantly, our lipidomics analysis revealed that Cd exposure induces lipid metabolism disorders. The levels of DALs were significantly changed, with a significant enrichment of the glycerophospholipid metabolic pathway. This is consistent with previous findings that heavy metal contamination disrupts normal metabolic processes, including lipid metabolism [41,42]. Our results revealed that changes in lipid metabolism may be closely related to the pathogenesis of intestinal injury in Cd-exposed to Hu Sheep. Lipids play an important role in maintaining cell membrane integrity and energy metabolism. Disturbances in lipid metabolism can lead to abnormal cellular functions and trigger inflammation and tissue damage [43,44].

In our study, sodium octanoate, a medium- and long-chain fatty acid derivative, emerged as a potential therapeutic agent against Cd-induced intestinal injury in Hu Sheep by reduced histopathologic scores, decreased levels of inflammatory cytokines, and restoration of the balance between pro-inflammatory and anti-inflammatory cytokines (IL-17 and IL-10). Sodium octanoate also repaired the disrupted intestinal barrier integrity, increased the number of MUC2-positive cells, and reversed abnormal genes expression associated with oxidative stress [22]. In terms of lipid metabolism, SO alleviated lipid metabolism disorders induced by Cd exposure, restored levels of key lipid metabolites, and normalized the glycerophospholipid metabolic pathway.

AMP-Activated protein kinase, a central energy sensor maintaining cellular metabolic homeostasis, plays a crucial role in lipid metabolism by inhibiting lipid synthesis and promoting lipolysis and oxidation [45,46]. The key role of AMPK in the regulation of lipid metabolism has been confirmed by many research results. For example, it has been found that Naringenin can effectively inhibit lipid accumulation by activating the AMPK pathway, and Gastrodin can also significantly reduce the lipid accumulation phenomenon in non-alcoholic steatohepatitis (NASH) by activating AMPK [47,48]. Our results showed that Cd exposure activated the AMPK signaling pathway through the *LKB1-STRAD-MO25* complex, and SO treatment reversed the gene expression changes associated with AMPK pathway. Sodium octanoate partially restored the dynamic balance of lipid metabolism and promoted the repair of intestinal damage.

In addition, comprehensive lipidomic and transcriptomic analyses revealed a significant correlation between key regulatory genes of the AMPK signaling pathway and specific phospholipid species. This correlation further revealed a potential link between lipid metabolism and transcriptional regulation, suggesting a mechanism for their synergistic action in Cd induced intestinal injury and the therapeutic effects of SO. For example, our study found significant correlations were found between some phospholipids and inflammatory cytokines, oxidative stress indicators, and lipid synthases, suggesting that they may play an important bridging role in the regulation of intestinal inflammatory responses, oxidative stress states, and lipid metabolism. We also found that Cd exposure led to the down-regulation of genes related to glycerophospholipid synthesis (e.g., *PCYT1A*, *PCYT1B*), and the aberrant expression of these genes disrupted the integrity and fluidity of membrane lipid rafts. Sodium octanoate treatment significantly restored the expression of these genes, which further confirmed that the AMPK pathway may be involved in the repair process of Cd toxicity through the regulation of membrane lipid synthesis. However, further in-depth studies on the specific molecular mechanisms of the regulation of the expression of these genes, as well as their interactions with other intracellular signaling pathways, are still needed.

The present study provides new insights into understanding the effects of heavy metal pollution on intestinal health and lipid metabolism in livestock, as well as a theoretical basis and potential therapeutic targets for the development of preventive and control strategies against Cd-induced intestinal damage. Future studies could be further extended to different breeds of livestock and a wider range of heavy metal pollutants to deeply investigate the general applicability and mechanism of action. In addition, the combination of other emerging technologies, such as single-cell sequencing and proteomics, will help to reveal more comprehensively the molecular mechanisms of Cd-induced intestinal injury and the deeper principles of the therapeutic effects of SO, providing stronger support for healthy livestock breeding and environmental protection.

## 5. Conclusions

In this study, we delved into the effects of Cd exposure on the intestines of Hu Sheep and the therapeutic role of SO, providing key insights into coping with intestinal damage in livestock due to Cd contamination. The results showed that Cd exposure triggered weight loss, intestinal inflammation, impaired barrier function, oxidative stress imbalance, and lipid metabolism disorders in Hu Sheep. These adverse effects are a serious threat to the health and growth performance of Hu Sheep, with potential losses to the livestock industry. Notably, SO demonstrated significant effects in ameliorating Cd induced intestinal damage. It was able to reduce intestinal inflammation, repair the damaged intestinal barrier, restore immune homeostasis, and alleviate lipid metabolism disorders. Specifically, SO effectively ameliorates lipid metabolism abnormalities by modulating the glycerophospholipid metabolic pathway and normalizing the levels of key lipid metabolites. Further studies revealed that the protective mechanism of SO is closely related to the AMPK signaling pathway. Cadmium exposure over-activated the AMPK signaling pathway, whereas SO treatment reversed the changes in the expression of related genes, restored the dynamic balance of lipid metabolism, and then promoted the repair of intestinal damage. This suggests that SO may exert its protective effect against Cd induced intestinal injury by regulating the activity of AMPK signaling pathway. This finding provides a novel strategy and direction for the prevention and treatment of Cd pollution-mediated intestinal diseases in livestock, and is expected to provide strong support for solving the heavy metal pollution challenges faced by the animal husbandry industry.

## Data Availability

The experimental datasets supporting this study are available upon request through the corresponding author.

## Supplementary Materials

The following supporting information can be downloaded at: https://www.mdpi.com/article/10.3390/biom15050707/s1, Figure S1. (A) PRKAG1, PRKAG2, PRKAG3, CPT1 and MCD gene expression levels in the three groups. (B) CDS2, LPIN1, LPIN2, CEPT1 and LPCAT2 gene expression levels in the three groups. All the above experiments were repeated three times independently and the data were expressed as “Mean ± standard deviation (SD)”. * *p* < 0.05, ** *p* < 0.01, *** *p* < 0.001. Table S1. Primary antibody for immunostaining. Table S2. RT-qPCR Primers Sequences.
