# Supplementary material for: AMPK Signaling Axis-Mediated Regulation of Lipid Metabolism: Ameliorative Effects of Sodium Octanoate on Intestinal Dysfunction in Hu Sheep
Source: Biomolecules. 2025 May 12;15(5):707. doi: 10.3390/biom15050707 (PMC12108909; doi:10.3390/biom15050707)
Supplement: Supplementary file 1 [file biomolecules-15-00707-s001.zip › biomolecules-3608844-supplementary.pdf]

Supplementary Tables and Figures

Table S1. Primary antibody for immunostaining

Name	Dilution ratio
anti-MUC2	1:2000
Ki67	1:200
IL-17	1:200
IL-10	1:200
Claudin 1	1:200
β-catenin	1:200
CD4	1:200
CD8	1:200

Table S2. RT-qPCR Primers Sequences

Name	Sequence (5' to 3')
18SRNA-forward	GTAACCCGTTGAACCCCATT
18SRNA-reverse	CCATCCAATCGGTAGTAGCG
LKB1-forward	CACCCGTTTGCCGAGGATGAC
LKB1-reverse	CAGATGTCCACCTTGAAGCCAGAG
CPT1-forward	CGGTTGCTGATGACGGCTATGG
CPT1-reverse	TCCCGAAGCGATGCGAGTCC
PRKAB2-forward	CACTGTTATCCGCTGGTCTGAAGG
PRKAB2-reverse	GAGGTCCAGGATGGCAACAAAGTC
SCD1-forward	CCACAACTACCACCACACCTTTCC
SCD1-reverse	ACGGCAGCCTTGGATACTTTCTTC
FAS-forward	GACCTTTCCAACAACCACCCTCTG
FAS-reverse	CCTTCAGCAGCGATGACACTTCC
SREBP1c-forward	CTCCGACACCACCAGCATCAAC

SREBP1c-reverse	GCAGCCCATTCATCAGCCAGAC
MCD-forward	GCTACTTCTTCTCTCACTGCTCAAC
MCD-reverse	ATCGTCTGGATGCTGCTGGAG
STRAD-forward	ACACAAGTTGAATCACTCAGACCTG
STRAD-reverse	GGATGAAGGCAGCAAGGAAAGG
MO25-forward	TCCTCCTCAAGAACCAAGCCAAG
MO25-reverse	CTCATCATTGAACTGCTCGTCCTC
ACC1-forward	GCTATGGAAGTCGGCTGTGGAAG
ACC1-reverse	TCGTCAGGAAGAGGCGGATGG
PRKAG1-forward	GGTGGATGAGAAAGGGCGTGTG
PRKAG1-reverse	TGATCGGTGTTGCAGGGCTTTG
PRKAG2-forward	CATCGGCTGGTGGTCGTCAATG
PRKAG2-reverse	CGGCTTCTGTTTGCTTCTGTTTGG
PRKAG3-forward	GCCTCTACTCCCGCTTTGATGTG
PRKAG3-reverse	TCCTCCGCTTCAGTGCTTCTCC

Fig S1

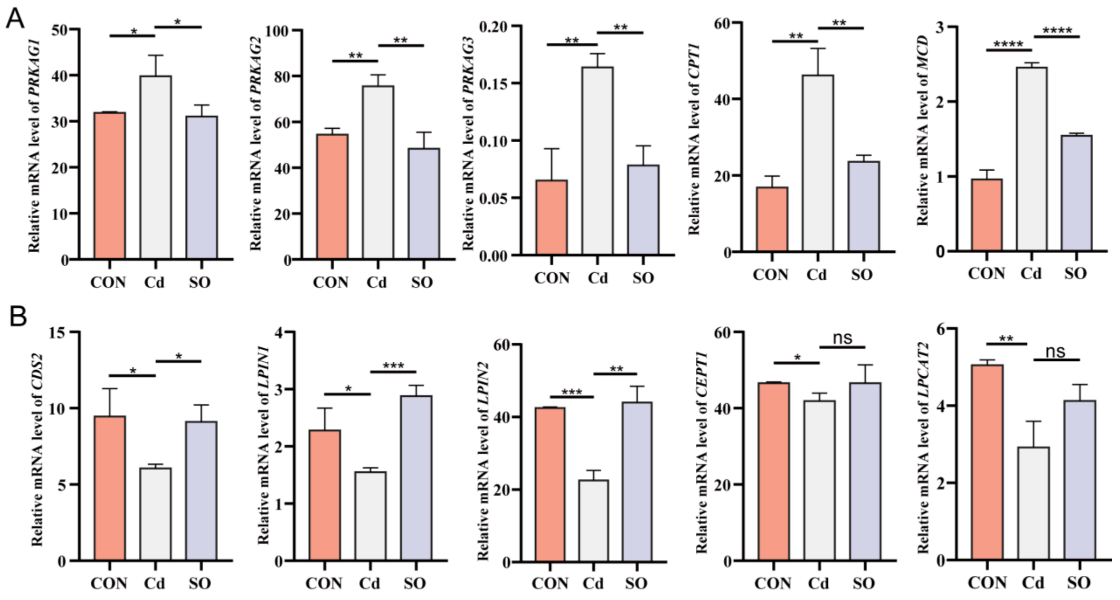

Figure S1. (A) *PRKAG1, PRKAG2, PRKAG3, CPT1* and *MCD* gene expression levels in the three groups. (B) *CDS2, LPIN1, LPIN2, CEPT1*

and *LPCAT2* gene expression levels in the three groups. All the above experiments were repeated three times independently and the data were expressed as "Mean ± standard deviation (SD)". $^{*}P < 0.05$, $^{**}P < 0.01$, $^{***}P < 0.001$.
